# Golgi localisation of GMAP210 requires two distinct cis-membrane binding mechanisms

**DOI:** 10.1186/1741-7007-7-56

**Published:** 2009-08-28

**Authors:** Jesus Cardenas, Sabrina Rivero, Bruno Goud, Michel Bornens, Rosa M Rios

**Affiliations:** 1 CABIMER-CSIC, Seville, Spain; 2 UMR144 CNRS-Institut Curie, Paris, France

## Abstract

**Background:**

The Golgi apparatus in mammals appears as a ribbon made up of interconnected stacks of flattened cisternae that is positioned close to the centrosome in a microtubule-dependent manner. How this organisation is achieved and retained is not well understood. GMAP210 is a long coiled-coil cis-Golgi associated protein that plays a role in maintaining Golgi ribbon integrity and position and contributes to the formation of the primary cilium. An amphipathic alpha-helix able to bind liposomes *in vitro *has been recently identified at the first 38 amino acids of the protein (amphipathic lipid-packing sensor motif), and an ARF1-binding domain (Grip-related Arf-binding domain) was found at the C-terminus. To which type of membranes these two GMAP210 regions bind *in vivo *and how this contributes to GMAP210 localisation and function remains to be investigated.

**Results:**

By using truncated as well as chimeric mutants and videomicroscopy we found that both the N-terminus and the C-terminus of GMAP210 are targeted to the cis-Golgi *in vivo*. The ALPS motif was identified as the N-terminal binding motif and appeared concentrated in the periphery of Golgi elements and between Golgi stacks. On the contrary, the C-terminal domain appeared uniformly distributed in the cis-cisternae of the Golgi apparatus. Strikingly, the two ends of the protein also behave differently in response to the drug Brefeldin A. The N-terminal domain redistributed to the endoplasmic reticulum (ER) exit sites, as does the full-length protein, whereas the C-terminal domain rapidly dissociated from the Golgi apparatus to the cytosol. Mutants comprising the full-length protein but lacking one of the terminal motifs also associated with the cis-Golgi with distribution patterns similar to those of the corresponding terminal end whereas a mutant consisting in fused N- and C-terminal ends exhibits identical localisation as the endogenous protein.

**Conclusion:**

We conclude that the Golgi localisation of GMAP210 is the result of the combined action of the two N- and C-terminal domains that recognise different sub-regions of the cis-GA. Based on present and previous data, we propose a model in which GMAP210 would participate in homotypic fusion of cis-cisternae by anchoring the surface of cisternae via its C-terminus and projecting its distal N-terminus to bind the rims or to stabilise tubular structures connecting neighbouring cis-cisternae.

## Background

In mammalian cells, the Golgi apparatus (GA) is composed of stacks of cisternae laterally linked by tubules to create a membrane network, the Golgi ribbon, whose formation depends on its unique pericentrosomal position [1,2]. Microtubules (MTs) play an important role in maintaining integrity and positioning of the Golgi ribbon, which is severely altered when MTs are depolymerised or when minus-end directed motors are inactivated [3-5]. Maintaining the architecture of the Golgi ribbon also requires continuous input of membranes from the ER [6] and regulated lateral fusion of analogous cisternae [7]. Recent work has begun to identify components necessary for linking cisternal stacks into a contiguous Golgi ribbon [6-8]. Depletion of Golgi associated proteins such as Golgin160 or GMAP210 has been shown to also disrupt formation of the Golgi ribbon [9].

GMAP210 was first identified as a cis-Golgi associated protein that redistributed to the intermediate compartment (or to ER exit sites) in the presence of Brefeldin A (BFA) [10]. We and others have shown that GMAP210 co-sedimented with taxol-purified MTs [11,12] and its over-expression profoundly perturbed, not only GA structure and function, but also MT-network organisation [12]. We further reported that GMAP210 co-precipitated γ-TuSC and was able to recruit γtubulin to Golgi surface in a C-terminus-dependent manner. When targeted to the mitochondria surface, GMAP210 induced their redistribution to a pericentrosomal location [13]. In addition, GMAP210 depletion by siRNA resulted in extensive fragmentation of the GA. Based on these data, we proposed that by combining MT-anchoring and membrane fusion activities GMAP210 contributes to Golgi ribbon formation around the centrosome [13]. We also identified a Golgi-targeting signal in the N-terminus of GMAP210. However, others failed to observe Golgi localisation of the N-terminus but identified a Grip-related Arf-binding domain (GRAB domain) at the C-terminus that interacts with Golgi membranes [14]. Mutation of a leucine residue predicted to be critical for GRAB-Arf binding abolished GA recruitment of the C-terminal domain of GMAP210. However, the effect of this mutation on the full-length protein localisation was not investigated. In addition, interaction with Arf1 has only been demonstrated *in vitro *[15] and, the behaviour of GMAP210 in response to BFA is not compatible with a simple Arf1-dependent Golgi association. The existence of two Golgi-targeting motifs in GMAP210 sequence has been recently confirmed [16].

A bioinformatics search has recently revealed the presence of an ALPS (amphipathic lipid-packing sensor) motif at the first 38 residues of GMAP210 [17]. The ALPS motif, a lipid-binding module first identified in ArfGAP1 [18], is unstructured in solution but folds into an α-helix once bound to highly curved membranes. Because of this behaviour, this motif is considered as a membrane curvature sensor. An analysis of attachment properties of GMAP210 N- or C-termini to liposomes revealed different requirements: N-terminus preferably bound to small liposomes whereas the C-terminus was recruited to liposomes in an Arf1-GTP-dependent, size-independent manner. Since ArfGAP1 displays curvature-dependent ArfGAP activity *in vitro*, it would be expected that Arf1-GTP is confined to flat membranes where ArfGAP activity is rather low. Accordingly, addition of recombinant Arf-GAP1 decreased the association of the GMAP210 C-terminus to small liposomes [15]. Based on these data, and on fluorescence and electron microscopy, a model was proposed in which by tethering positively curved membranes via its N-terminus to flat membranes via its C-terminus GMAP210 could serve to capture small transport vesicles in the interface ER-Golgi. Other studies have recently reported that Arf1-GTP is able to induce positive curvature through the insertion of its N-terminal amphipathic helix in the lipid bilayer, in a similar manner to the small GTPase Sar1p. In this latter model, Arf1-GTP would be membrane-associated in positive-curvature regions where it could recruit effector molecules [19-21].

The mechanism of membrane binding based on the detection of membrane curvature is likely to be relevant to GMAP210 function. However, the physiological pathway in which it is used remains to be characterised. Immunolectron microscopy has revealed enrichment of GMAP210 in curved regions of the cis-Golgi but has not revealed binding of GMAP210 to any type of vesicles either in mammalian [10,22] or in *Drosophila *cells [23]. Moreover, depletion of GMAP210 did not affect secretion in both systems [9,23]. On the contrary, overexpression of the protein profoundly disturbed Golgi structure, and Golgi proteins from different compartments including GMAP210 were found in vesicle clusters distributed throughout the cell. Under these conditions, anterograde and retrograde transport pathways were blocked [22,23] although this effect could be secondary to the Golgi structure perturbation.

Recent studies have identified new GMAP210 partners. Those include the small GTPase rab2 that interacts with dGMAP in a coiled-coil region close to the C-terminus [24] and the ciliary assembly protein IFT20. rab2 is localised to the cis-GA and has been found to bind MTs and to recruit dynein to membranes [25]. IFT20 is one of the Intraflagellar Transport Proteins required for the assembly of the primary cilium, and the only one that is localised to the GA, in addition to the centrosome and the cilium [26]. GMAP210 mutant mice are viable until birth, when they die from a pleiotrophic phenotype that includes growth retardation and lung and heart defects [16]. Those mice cannot be considered knockout mice *sensu stricto *since they express a truncated form of the protein consisting of the first 196 amino acids. This polypeptide includes the ALPS motif and therefore the possibility that it could interfere with membrane dynamics *in vivo *should be taken into account. Cells from GMAP210 mutant mice were able to form cilia but they were shorter than normal and contained less polycystin-2 than those detected in control cell lines. In mutant cells the IFT20 protein level is also reduced. Whether defects of GMAP210 mutant mice are due to a role of the protein in ciliary assembly through anchoring IFT20, as proposed, or to additional functions remain to be determined.

Finally, cells derived from GMAP210 mutant mice apparently exhibited a normal Golgi. This is in marked contrast with our previous results of RNAi-mediated inhibition of GMAP210 expression in HeLa cells [13] and with those recently published by the Linstedt group: in this study, GMAP210 was identified in a siRNA screeening as a component whose knockdown significantly fragmented and dispersed the GA [9]. Depletion of GMAP210 yielded a phenotype in which peri-centrosomal positioning of the GA was disrupted, the Golgi ribbon was fragmented into ministacks similar to those present in nocodazole-treated cells, but secretion exhibited normal kinetics. Strikingly, these cells completely failed to polarise and migrate in wound-healing assays. A role of GMAP210 in minus-end directed motility of Golgi membranes has been proposed.

All available data are consistent with GMAP210 having at least two membrane targeting motifs. However, how these motifs determine the GMAP210 localisation *in vivo *remain unclear. We have now performed a detailed analysis of Golgi binding properties of GMAP210 by generating a battery of truncated and fusion mutants. We have analysed their behaviour in non-transformed RPE1 cells under several conditions applying optical microscopy and video-recording approaches.

## Results

### The GMAP210 N-terminal domain is targeted to the cis-Golgi and the ERES

**Figure 1:**
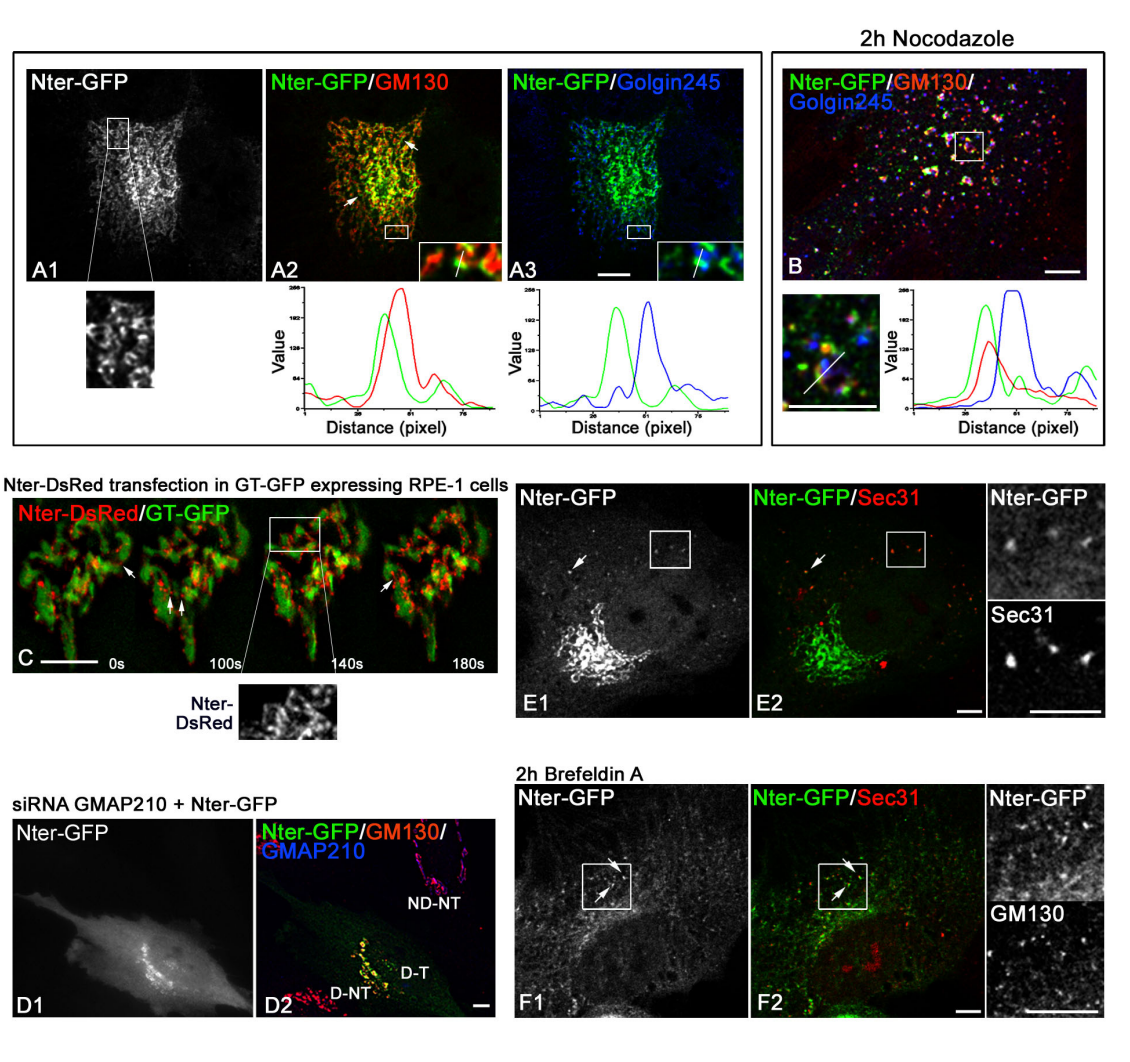
**The N-terminal domain of GMAP210 targets early compartments of the secretory pathway**. **(A) **Confocal images of RPE1 cells transfected with the Nter-GFP construct (A1) and double labelled for GM130 (A2, merged image) and Golgin245 (A3, merged image). An enlarged view of A1 is shown at the bottom. Insets show enlarged views. In bottom panels, fluorescence intensity profiles of lines drawn in insets are shown. **(B) **Nter-GFP transfected RPE1 cells treated with nocodazole and stained for GM130 and Golgin245. Bottom panels show an enlarged view (left) and fluorescence intensity profiles (right) of the line drawn over a nocodazole-induced Golgi element. **(C) **Dynamics of the NterDsRed fusion protein in a RPE1 cell line stably expressing GT™-GFP. Selected frames at the indicated times from Movie 1 (in Additional file 1) are shown. An enlarged view is shown at the bottom. Arrows point to sites in which the NterDsRed protein is located between two membrane elements. **(D) **RPE1 cells treated with siRNA against GMAP210 for 72 h were transfected with the Nter-GFP construction (D1), incubated for 16 h and then fixed and immuno-stained for GM130 in red and GMAP210 in blue (D2). ND-NT indicates a non-depleted-non-transfected cell, D-NT a depleted non-transfected cell, and D-T a depleted and transfected cell. **(E, F) **Nter-GFP transfected cells (E) were treated with BFA (F) and then stained for Sec31 to reveal ER exit sites. Enlarged views of individual labellings are shown at right. Arrows indicate ERES containing the GMAP210 N-terminal domain. Bars = 5 μm

As previously reported in other cell types, the N-terminal domain of GMAP210 in fusion with GFP (Nter-GFP, 1–375 aas) appeared Golgi-associated in RPE1 cells as revealed by double labelling for the cis-Golgi protein GM130, or the TGN-associated protein Golgin245 (Figure 1A). For analysis purposes, we selected cells with comparable low expression levels in all our experiments. High resolution images revealed that GFP labelling was not uniform and the fusion protein seemed to accumulate at specific sites (Figure 1A1, enlarged view). Fluorescence intensity profiles along lines drawn over the Golgi area showed the degree of co-localisation of each pair of proteins (Figure 1A, bottom panels). Nter-GFP co-distributed with GM130, but not with Golgin245, indicating that the N-terminus of GMAP210 specifically targets the cis-side of the GA. The cis-Golgi localisation of the Nter-GFP protein was preserved after nocodazole-induced disruption of the Golgi ribbon (Figure 1B) indicating that targeting of the N-terminal domain to the cis-Golgi does not depend on Golgi ribbon integrity.

To address the dynamics of GMAP210 N-terminal domain in living cells, we generated a DsRed fusion protein (Nter-DsRed) and transfected it in an RPE1 cell line stably expressing the transmembrane domain of galactosyltransferase fused to GFP (GT™-GFP; see also Additional file 1). A spotty distribution of the Nter-DsRed fusion protein at the periphery of Golgi elements could be appreciated (Figure 1C). In addition, it appeared enriched in regions that seem to bridge two adjacent elements (Figure 1C, arrows). Note that DsRed-tagged N-terminal domain, like Nter-GFP fusion protein, unevenly distributed on Golgi membranes (compare enlarged views of Figures 1A1 and 1C).

Since GMAP210 dimerise [27], we wanted to exclude the possibility that Golgi binding was due to association with endogenous protein. To do this, we transfected the Nter-GFP construct in GMAP210-silenced RPE1 cells by siRNA. Decreasing concentrations of GMAP210 in RPE1 cells fragmented the Golgi ribbon as in HeLa cells although elements remained closer to one another. Figure 1D shows a non-depleted non-transfected (ND-NT), a depleted nontransfected (D-NT) and a depleted transfected (D-T) cells. As can be seen, the Nter-GFP protein was recruited to Golgi elements even in the absence of endogenous GMAP210.

Interestingly, some dots scattered throughout the cytoplasm that co-localised with Sec31 were also observed in most cells, indicating that the Nter-GFP was also able to bind ER exit sites (ERES; Figure 1E, arrows). In addition, it re-localised to ERES upon BFA treatment (Figure 1F), a behaviour that is reminiscent of that of the endogenous GMAP210 protein [10]. Altogether, these data demonstrate that GMAP210 N-terminal domain binds directly to membranes *in vivo*, specifically targets the cis-Golgi and can be recruited to ERES both in non-treated and in BFA-treated cells.

### The N-terminal cis-Golgi localisation signal corresponds to the ALPS motif

**Figure 2:**
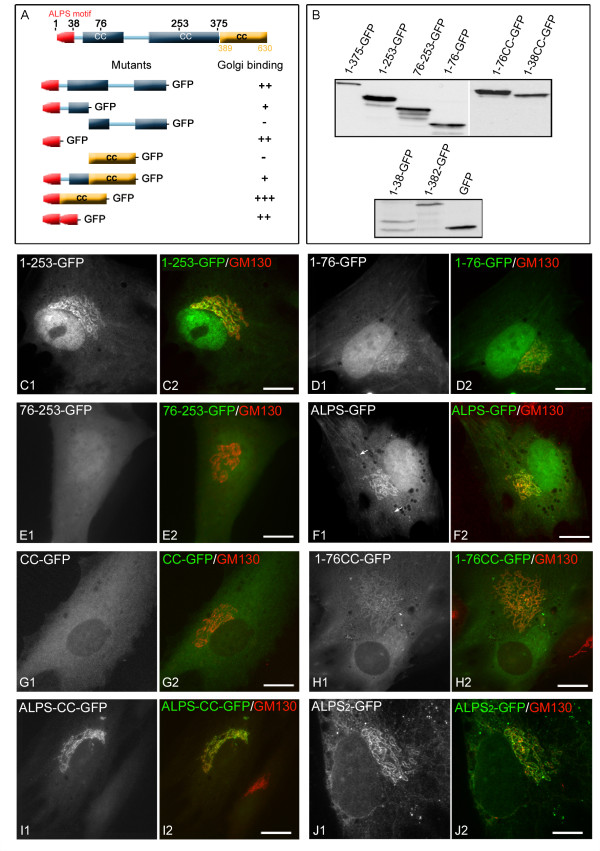
**The ALPS motif of GMAP210 is sufficient for Golgi binding**. **(A, B) **Schematic representation of the GFP-tagged N-terminal domain of GMAP210 and all the truncated or fusion constructs in this domain that were tested for their binding to GA (A) and their migration on SDS-PAGE as revealed by Western blot with an anti-GFP antibody after expression in RPE1 cells (B). The ALPS-like motif is represented in red and coiled-coil regions in light blue. The yellow rectangle corresponds to the continuous coiled-coil domain used in the indicated constructs. **(C to J) **Subcellular localisation of the different N-terminal mutants and other constructs (as indicated) expressed in RPE1 cells and double labelled for GM130. Arrows in (F1) indicate ERES. Bars = 10 μm.

To define the module responsible for N-terminal domain recruitment to membranes, we generated additional truncated forms as shown in Figure 2A. The ALPS-like motif is represented in red and coiled-coil regions in light blue. The yellow rectangle corresponds to the continuous coiled-coil domain used in the indicated constructs. Western blot analysis of all truncated mutants revealed bands with apparent sizes matching the predicted sizes (Figure 2B).

**Figure 3:**
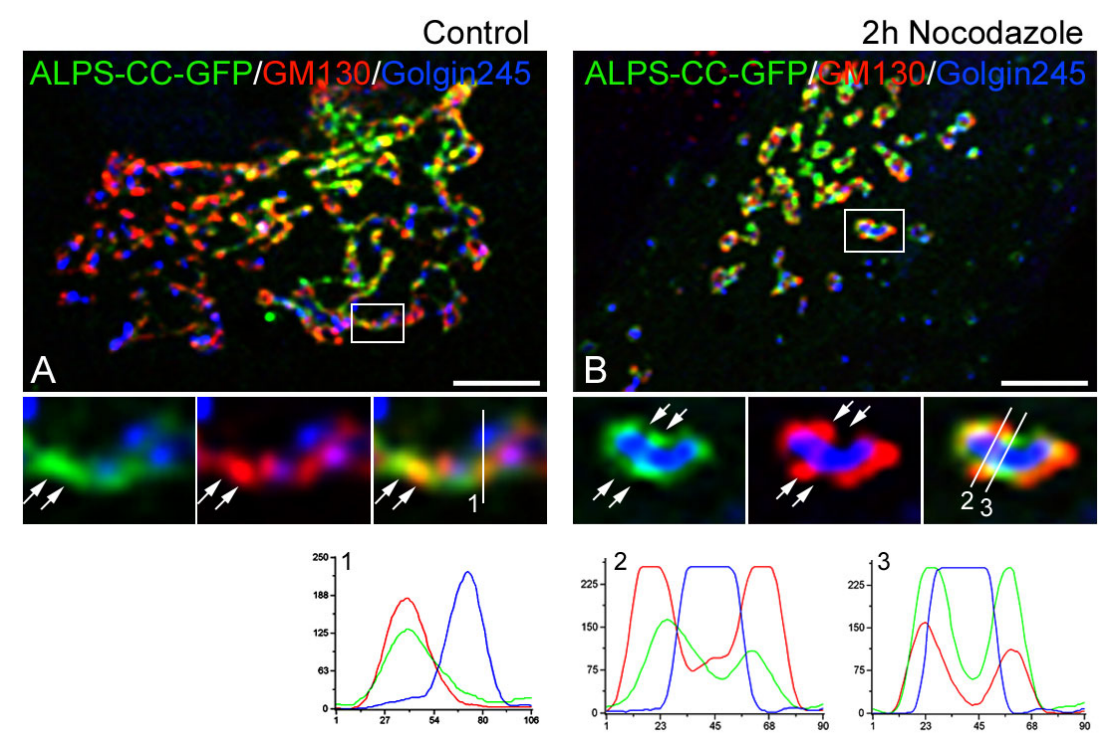
**A detailed analysis of the distribution of the ALPS-CC mutant protein in Golgi membranes**. **(A, B) **Cells transfected with the ALPS-CC-GFP construct (A) were treated (B) with nocodazole and labelled with anti-GM130 and anti-Golgin245 antibodies. Merged images are shown. Enlarged views of insets show merged images of GFP and Golgin245 labellings in left panels, merged images of GM130 and Golgin245 labellings in middle panels and merged images of the three labellings in right panels. At the bottom, fluorescence intensity profiles of lines drawn over Golgi membranes revealed cis-Golgi localisation of the mutant (lane 1) but also a striking enrichment in regions from which GM130 is excluded and vice versa (arrows and lanes 2 and 3). Bars = 5 μm.

The 1-253-GFP exhibited identical GA localisation to the whole N-terminal domain (1-375-GFP) (Figure 2C; compare with Figure 1A). A construct containing only the first 76 amino acids weakly associated with GA (Figure 2D) whereas no GA targeting was observed with the complementary mutant (76-253-GFP; Figure 3E). Finally, the first 38 amino acids of the protein, corresponding to the ALPS-like motif, displayed good Golgi localisation that coincided with GM130 (Figure 2F) and also decorated some ERES (arrows in Figure 2F1). Surprisingly, this construct showed a stronger Golgi binding than the 1-76-GFP one (compare Figure 2D and Figure 2F; see below), suggesting the presence of inhibitory sequences downstream of the amphipathic helix.

We further examined the capacity of the ALPS-like motif to target another polypeptide to GA *in vivo*, namely a segment of GMAP210 corresponding to a predicted continuous coiled-coil with maximum probability to dimerise (amino acid 389–630), that did not apparently contain any targeting signal and was excluded from the nucleus (Figure 2G). Two chimeric constructs, named 1–76CC and ALPS-CC respectively, were expressed in fusion with GFP. Addition of the coiled-coil fragment significantly improved GA association of the ALPS sequence (Figure 2I) but not that of the 1–76 one (Figure 2H). Quantification of these experiments indicated that the affinity for Golgi membranes of the ALPS-CC construct is 2.8 times higher than of the 1–76CC fusion protein, which supports the possibility of an inhibitory activity of aminoacids 38–76.

Strikingly, ALPS-CC showed a remarkably higher GA specificity than all the other constructs including the whole N-terminal domain (compare Figure 1A and Figure 2I). The construct was exclusively targeted to the GA and, in fact, the distribution pattern of ALPS-CC was very similar to that of the endogenous protein. The most likely explanation is that the CC segment mediates the dimerisation of the fusion protein, mimicking the structure of the wild-type protein and increasing the avidity of the interaction and the selectivity for cis-Golgi membranes. To support this view we designed an additional construct in which two ALPS-like motifs were placed in tandem separated by a short break. As expected, this significantly increased GA association (Figure 2J). These results conclusively demonstrate that the ALPS motif is able to bind GA membranes in the absence of any other targeting signal.

The remarkable specificity of the ALPS-CC construct prompted us to analyse more closely the membrane-binding properties of this polypeptide. We compared the distribution of this chimeric protein with respect to that of GM130 and Golgin245, in control (Figure 3A) or nocodazole-treated (Figure 3B) cells. In enlarged views of insets, pairs of labellings are as follows: GFP/Golgin245 labellings in left panels, GM130/Golgi245 labellings in middle panels, triple labellings in right panels. Fluorescence intensity profiles of lane 1 drawn over the Golgi area in control cells show that the chimeric protein is specifically targeted to the cis-Golgi (Figure 1A, bottom panel). A careful examination revealed, however, that it was unevenly distributed along the cis-side. GFP labelling appeared concentrated in regions in which the intensity of GM130 labelling was rather low. This was even more evident in individual Golgi ministacks generated by disruption of MTs (Figure 3B). An inverse correlation between GFP and GM130 labellings in fluorescence intensity profiles was noted (lanes 2 and 3).

We conclude that the ALPS-like motif provides GMAP210 with a mechanism to specifically recognise and bind membranes located at the cis-side of the GA. In addition, it is specifically enriched in areas in which other membrane-associated proteins such as GM130 are not present (see below).

### The C-terminal domain binds both cis-GA and centrosome

**Figure 4:**
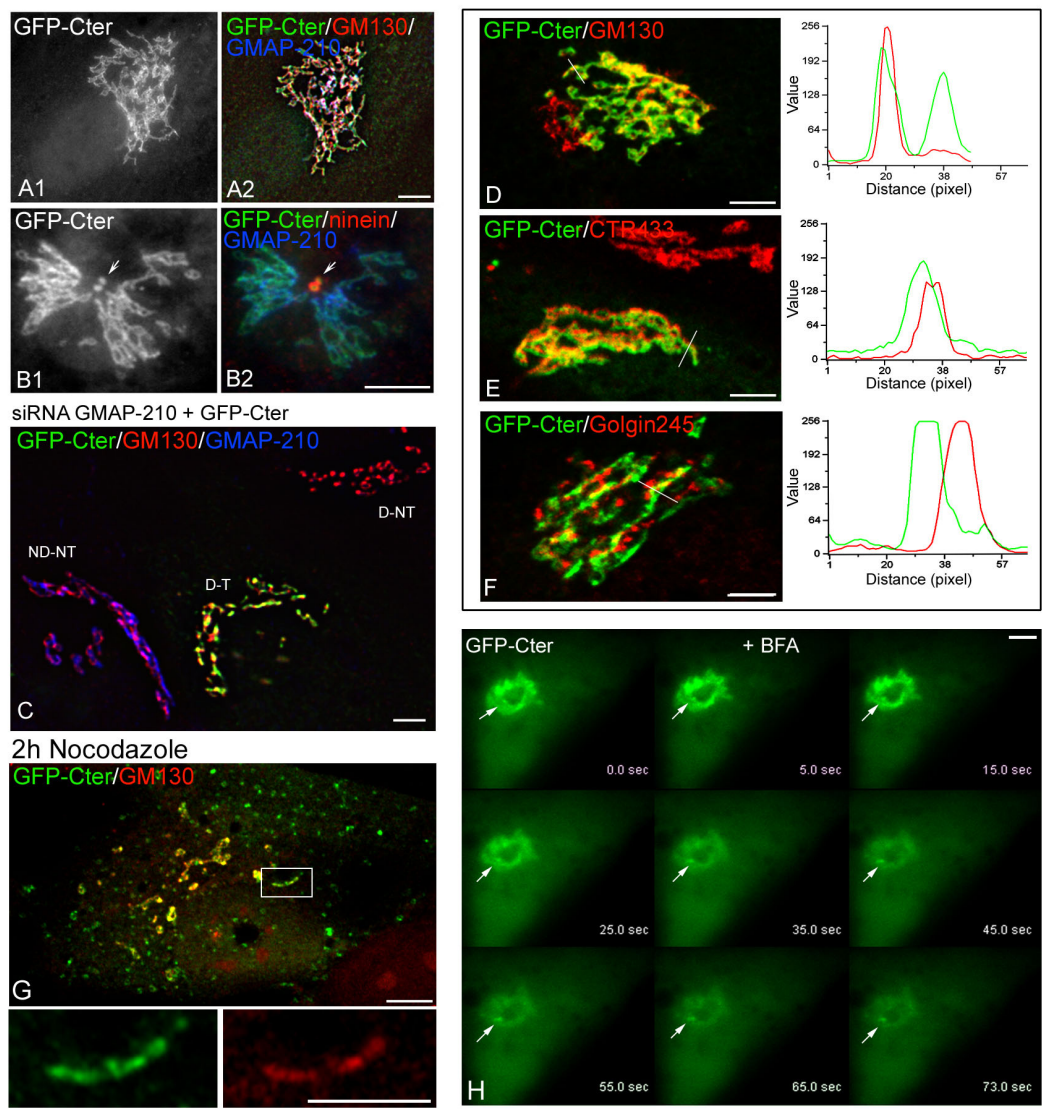
**The C-terminal region of GMAP210 targets the cis-Golgi compartment and the centrosome**. **(A, B) **RPE1 cells were transfected with the C-terminal domain of GMAP210 (aa 1778–1979) in fusion with GFP, fixed and double labelled for GM130 and endogenous GMAP210 (A) or for the centrosomal protein ninein and GMAP210 (B). Merged images are shown in A2 and B2. Arrows indicate the centrosome. **(C) **A merged image of GMAP210-depleted cells transfected with the GFP-Cter construct and stained for GM130 and endogenous GMAP210. ND-NT indicates a non-depleted non-transfected cell, D-NT a depleted non-transfected cell and D-T, a depleted and transfected cell. **(D to F) **Confocal images showing the distribution of GFP-Cter fusion protein with respect to the cis-Golgi marker GM130 (D), the medial Golgi marker CTR433 (E) or the trans-Golgi marker Golgin 245 (F). Corresponding fluorescence intensity profiles are shown at right. **(G) **A GFP-Cter transfected cell treated with nocodazole and labelled for GM130. Enlarged views of single labellings are shown at the bottom. **(H) **Live imaging of a GFP-Cter transfected cell treated with BFA. BFA was added to a final concentration of 2.5 μg/ml at time 5.0 seconds and images were taken with 0.2-second intervals. Selected frames of Movie 2 (in Additional file 2) at indicated time points are shown. Note that GFP-Cter rapidly dissociated from membranes after BFA addition whereas fluorescence at the centrosome decreased only slightly (arrow). Bars = 5 μm.

The binding properties of the GMAP210 C-terminal domain (aas 1778–1979) have also been investigated. Contrary to our previous results obtained in cold-methanol COS fixed cells [12], PFA-fixed RPE1 cells displayed a conspicuous GFP-labelling co-localising with GM130 and endogenous GMAP210 at the GA (Figure 4A); in both methanol and PFA-fixed cells an association with the centrosome was detected (Figure 4B, arrows). Association of the endogenous protein with the centrosome was not observed, confirming our previous data on COS7 cells [12]. Identical results were obtained when GFP-Cter was transfected in GMAP210-silenced cells (Figure 4C). Strikingly, confocal microscopy using markers of the cis-Golgi (GM130; Figure 4D), the medial-Golgi (CTR433; Figure 4E) or the trans-Golgi network (Golgin-245; Figure 4F) revealed also a specific recruitment of GFP-Cter to the cis face of GA. In marked contrast with the N-terminal domain, however, GFP-Cter distribution was continuous along the ribbon.

When cells expressing the GFP-Cter construct were treated with nocodazole, centrosomal GFP labelling disappeared (not shown) whereas labelling remained on the scattered Golgi elements (Figure 4G). To investigate the effects of BFA on GFP-Cter membrane association, we employed a time-lapse microscopy approach (Figure 4H; see also Additional file 2). GFP-Cter rapidly dissociated from membranes after BFA addition. Since BFA is an inhibitor of Arf-specific guanine nucleotide exchange factors which prevents GTP loading of Arf1, the behaviour of the C-terminal domain supports the possibility that GMAP210 could be an ARF1 effector (see below). On the contrary, centrosomal labelling remained (arrow), suggesting that molecular mechanisms mediating the association of this domain with GA or centrosome are distinct.

### Membrane binding motifs of GMAP210 distribute differentially within GA

**Figure 5:**
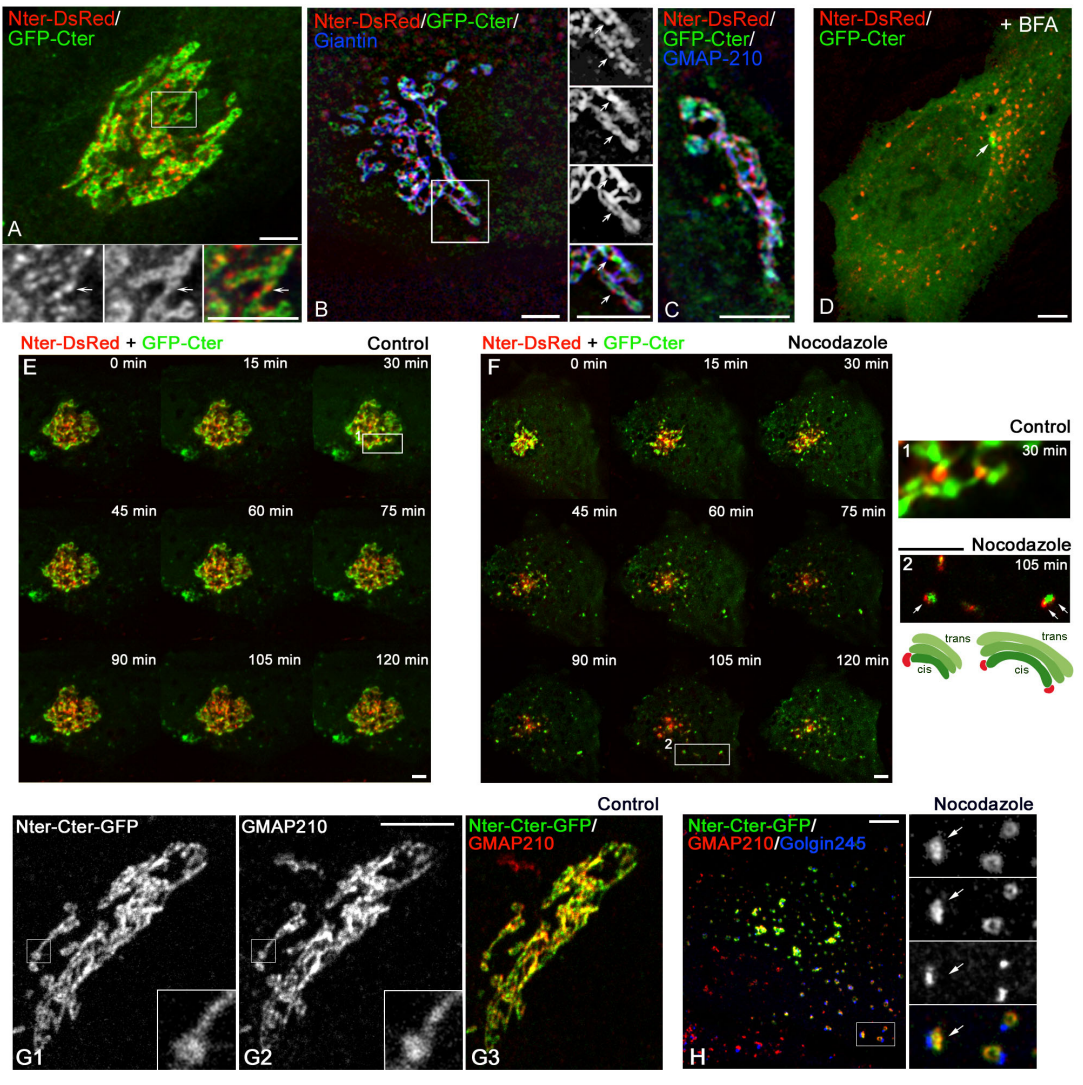
**The N-terminus and the C-terminus of GMAP210 bind different regions of the Golgi ribbon**. **(A to C) **Nter-DsRed (1–375 aas) and GFP-Cter (1778–1979 aas) constructs were co-transfected in RPE1 cells and their distribution analysed by confocal microscopy (A) or deconvolution (B, C). Merged images of the two constructs (A) or of the two constructs with giantin (B) or with endogenous GMAP210 (C) immunolocalisation are shown. High magnifications of the selected regions in (A) and (B) show individual labellings and the merge. Arrows indicate identical locations for each panel. **(D) **RPE1 cells co-transfected with Nter-DsRed and GFP-Cter and treated with BFA. All GFP-Cter becomes cytosolic except at the centrosome (arrow). **(E, F) **Time-lapse analysis of RPE1 cells transiently transfected with Nter-DsRed and GFP-Cter in control conditions or after nocodazole treatment. Selected frames of Movies 3 (control) or 4 (nocodazole) (see Additional files 3 and 4, respectively) are shown at the same time points. At right, enlarged views of selected areas are shown. **(G) **RPE1 cells transfected with a truncated mutant consisting of both N- and C-termini expressed in fusion (Nter-Cter-GFP, G1) and double labelled for endogenous GMAP210 (G2). A merged image is shown in G3. In **(H)**, a Nter-Cter-GFP transfected cell was treated with nocodazole and triple labelled for endogenous GMAP210 and Golgin245. High magnifications of the selected region at right show individual labellings and the merge. Arrows indicate identical localisation of the truncated mutant and the endogenous protein in isolated Golgi ministacks. Bars = 5 μm.

Cells were then co-transfected with Nter-DsRed and GFP-Cter constructs and further fixed, labelled and analysed by confocal microscopy (Figure 5A) or conventional immunofluorescence (IF) followed by deconvolution (Figures 5B and 5C). Only cells expressing low levels of both constructs were analysed. While both domains specifically target the cis-GA, a clear segregation of both constructs within GA was apparent (inset in Figure 5A) with the N-terminal domain enriched in areas that seem to connect adjacent elements (arrows). Segregation was also observed with respect to giantin (Figure 5B) and, more interestingly, to endogenous GMAP210 that remained associated to Golgi membranes even in the presence of both N- and C-terminal ends (Figure 5C). Treatment with BFA of double transfected cells confirmed the different redistribution of both ends of the protein: the C-terminal domain dissociated from membranes, but not from the centrosome, whereas the N-terminal domain redistributed to ERES (Figure 5D).

To trace the behaviour of GMAP210 terminal domains we performed time-lapse analysis of double transfected RPE1 cells (Figures 5E and 5F; see also Additional files 3 and 4). We selected cells expressing similar levels of both truncated mutants. Strikingly, in a 120 min interval of observation, the GA of control cells appeared unperturbed and rather static (Figure 5E). As noted above, neither separately nor simultaneously expressed, the terminal domains displaced the endogenous GMAP210 protein from membranes. The N-terminal domain mostly accumulated between membrane elements containing the C-terminal domain and seemed to bridge them in agreement with IF results (enlarged view 1 at right). After nocodazole addition, the GA fragmented and Golgi ministacks dispersed throughout the cytoplasm (Figure 5F). Individual Golgi ministacks contained both terminal domains but they did not co-localise: the N-terminal domain accumulated at the rims of the C-terminal containing cis-cisternae (enlarged view 2 at right).

Taken together, these results suggest that both ends of GMAP210 contribute to determine its localisation inside the GA. To definitively demonstrate this hypothesis we generated a new construct consisting of both terminal domains expressed in fusion (Nter-Cter-GFP). The distribution of this truncated mutant, which corresponds to the full-length protein lacking the long central coiled-coil, was almost identical to that of the endogenous GMAP210 in both control cells (Figure 5G) and isolated Golgi ministacks induced by nocodazole treatment (Figure 5H; arrows in enlarged view). We conclude that the localisation of GMAP210 is the result of the combined action of both N- and C-terminal ends.

### Both N-terminus or C-terminus are sufficient to target GMAP210 to the GA

**Figure 6:**
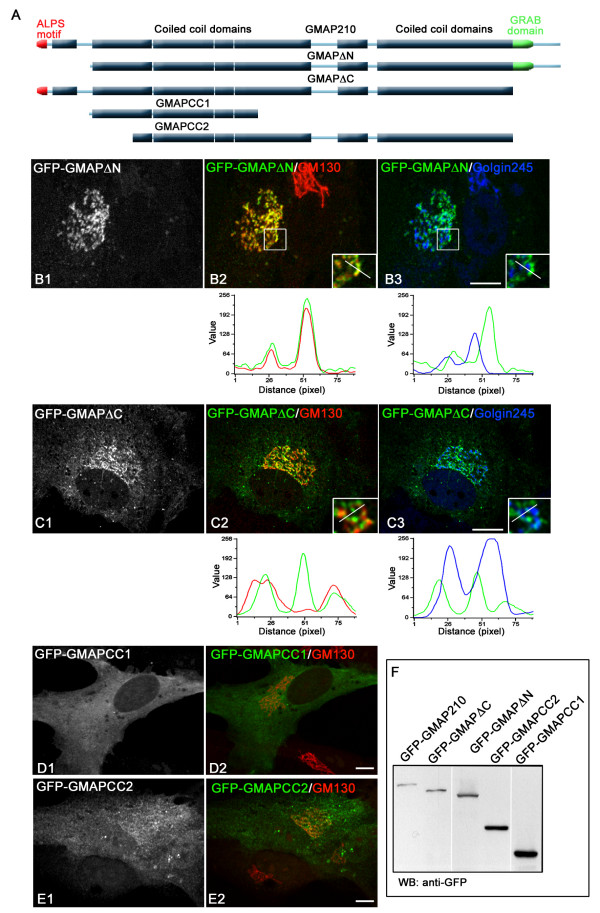
**Contribution of both Golgi binding domains to the localisation of GMAP210**. **(A) **Schematic representation of the full-length GMAP210 protein and of the truncated mutants used in this experiment. The ALPS-like motif is represented in red, coiled-coil regions in light blue, and the predicted GRAB domain in green. Western blot of all of these constructs expressed in RPE1 cells is presented in (**F**). **(B) **Cells expressing the mutant lacking the N-terminal domain (GFP-GMAPΔN (251–1979 aas)) were stained for GM130 (B2) and Golgin245 (B3). Insets show enlarged views. Fluorescence intensity profiles at the bottom revealed perfect co-localisation of this mutant with the cis-Golgi marker GM130. **(C) **Cells transfected with the mutant lacking the C-terminal domain (GFP-GMAPΔC (1–1778 aas)) were processed as above. Although the mutant mostly associated with the cis-side of the GA, it was also present in areas that did not contain GM130. In addition, it also accumulates in some tubular and vesicular structures devoid of GM130. **(D, E) **The central coiled-coil domains do not contain Golgi targeting information. Cells expressing the GFP-GMAPCC1 (D) or GFP-GMAPCC2 fusion proteins and labelled for GM130 are shown. Bars = 5 μm.

Finally, to evaluate the contribution of each membrane-binding domain to Golgi localisation of GMAP210, we generated GFP-fused truncated forms lacking either one or both, as represented in Figure 6A. Western blot analysis showed that proteins of expected size were expressed in transfected cells (Figure 6F). The mutant containing the C-terminus but lacking the N-terminus (GFP-GMAPΔN) accumulated at the GA as revealed by double labelling for the cis-Golgi protein GM130, or the TGN-associated protein Golgin245 (Figure 6B). Fluorescence intensity profiles along lines drawn over the Golgi area showed that GFP-GMAPΔN perfectly co-distributes with GM130, but not with Golgin245, indicating that the truncated mutant specifically binds the cis-side of the GA (Figure 6B, bottom panels). In the same way, the truncated mutant lacking the C-terminus (GFP-GMAPΔC) mostly localised to the GA (Figure 6C) although its distribution pattern was more complex. Similarly to the ALPS motif, it partially overlapped with GM130 and did not co-localise with Golgin245 (Figure 6C, bottom panels), indicating that it was also specifically targeted to the cis-side of the GA. In addition, the truncated mutant accumulated in some Golgi regions and in some peripheral spotty structures and tubules that did not contain GA markers. Finally, the long central coiled-coil domain, divided into two different fragments named CC1 (251–856) and CC2 (534–1779), did not apparently associate with any subcellular structure nor contribute to Golgi localisation in agreement with the behaviour of Nter-Cter-GFP fusion protein (Figures 6D and 6E).

## Discussion

Our present data demonstrate that the cis-Golgi localisation of GMAP210 involves two distinct membrane binding motifs located at the ends of the protein. Although both the N-terminal and the C-terminal domains specifically targeted the cis-GA, they did not co-localise nor did they with the endogenous protein. In addition, they did not displace the endogenous protein from the GA. However, when expressed in fusion as parts of the same polypeptide, it exhibited an almost identical localisation to the endogenous protein. Altogether these data support localisation of GMAP210 being the result of the combined action of the two ends of the protein that recognise different domains of the cis-GA.

The N-terminal Golgi binding sequence exactly matches the ALPS motif. This motif that has been reported to bind lipids and to act as a curvature sensor *in vitro *[17] exhibits a remarkable specificity *in vivo*: it accumulates at discrete regions of the cis-cisternae and also associates with some ER exit sites. Golgi binding significantly increased when the ALPS motif was expressed in a dimeric form either by expression in tandem (ALPS_2_-GFP) or by fusion with a non-adjacent coiled-coil segment with the highest probability to dimerise including a leucine zipper (ALPS-CC-GFP). The striking specificity of the latter construct (see Figure 3) indicates that the ALPS motif of GMAP210 is able to distinguish specific membranes among the bulk of intracellular membranes and, thus, to contribute to spatially restricting the protein inside the cell. This also suggests that high curvature cannot be the only parameter controlling its recruitment *in vivo *[17]. The ALPS motif was originally identified in the ArfGAP1 protein and proposed to interact with membranes by inserting hydrophobic residues into open spaces between lipid chains caused by the high curvature. Recently, it has been shown that ArfGAP1 actually contains two ALPS motifs that contribute to Golgi targeting [18,28-30]. From amino acid replacement experiments it has been proposed that moderate lipid disorder at the target membrane, such as that which may exist at the rims of the cisternae, would be enough for ALPS binding [30]. This is compatible with the distribution of the GMAP210 N-terminal domain at the periphery of NZ-induced Golgi ministacks (see Figure 5), and also with binding to membrane structures that connect Golgi stacks. In this regard, we have detected the presence of GMAP210 N-terminus in structures connecting membranes elements that contain: (i) the cis-Golgi protein GM130, (ii) the C-terminus of GMAP210 that was demonstrated to be evenly distributed throughout the cis-cisternae of Golgi stacks, (iii) the membrane-anchoring signal peptide of the galactosyltransferase that targets the GFP to the transmedial region of the GA. These data strongly support the possibility that the N-terminus of GMAP210 binds the connecting structures between Golgi stacks.

What is sensed by the ALPS motif in membranes besides lipid packing defects remains unknown, but the fact that it also localises to ER exit sites suggests a common feature. It has been reported that pre- and early GA compartments share a high content of PtdIns(4)P and low levels of cholesterol [31]. Localised PtdIns(4)P formation at ERES has also been described, supporting the possibility that ERES lipid composition resembles that of cis-Golgi rather than that of bulk ER [13,31,32]. Interestingly, GMAP210 *Drosophila *homolog appears associated with both cis-GA and ERES [23].

The full-length GMAP210 is known to redistribute at ERES upon BFA treatment like other cis-Golgi *matrix *proteins [10]. We have observed that all GA-associated ALPS-containing truncation mutants localised at ERES, not only upon BFA action but also in control conditions. On the contrary, mutants lacking this sequence, such as C-terminus-containing constructs, became cytosolic in the presence of BFA. Thus, the ability of ALPS motif to recognise ERES might be responsible for the redistribution of endogenous GMAP210 to these structures in response to BFA. Others have shown that a variety of cis-Golgi *matrix *proteins moved directly to ERES in the tip of BFA-induced membrane tubules [33]. The matrix proteins remained at and around the ER exit sites, whereas the trailing transmembrane proteins entered the ER at these sites and then redistribute throughout the ER. Our finding that GMAP210 ALPS motif associates with ERES in all conditions is the first hint as to how these retrograde membrane tubules could be targeted to ER membranes.

A second cis-Golgi binding signal is present in the C-terminal domain of GMAP210. Binding of the C-terminus to liposomes was recently shown to be dependent on ARF1-GTP and to involve the GRAB domain [15]. Since Arf1 localises throughout the GA stacks [34], specific recruitment of the GFP-Cter construct to the cis-GA *in vivo *was unexpected and suggests the involvement of additional factors that remain to be characterised.

In addition to the cis-GA, the GFP-C-terminal construct was targeted to the centrosome in RPE1 cells whereas the full-length protein has never been observed at the centrosome [12,13]. We hypothesised that the behaviour of this truncated mutant reflects its affinity for the minus ends of MTs. In support of this view, the C-terminal domain was able to displace the minus-end binding protein ninein from the centrosome, suggesting a competition of both proteins for MT minus ends (not shown), as previously reported with another centrosomal protein [12]. Moreover, we have found that RPE1 cells are unable to form aggresomes at the centrosome even after extensive treatment with the proteasome inhibitor MG132 (our unpublished data).

The GRAB domain was identified on the basis of its homology with the GRIP domain, an Arl1-interacting motif present in several coiled-coil proteins associated with the trans-GA. Interestingly, the subcellular distribution of the GRIP-containing protein Cbs in developing *Drosophila *varies during the cell division cycle from GA to chromosomes and centrosomes in the mitotic spindle [35]. This pattern dramatically changes in the absence of *Drosophila *Arl1 ortholog suggesting that localisation depends on the GRIP/Arl1 interaction. Indeed, a GFP-GRIP fusion peptide from Cbs was addressed to centrosomes and mitotic spindle and interfered with centrosome function. We have also observed that the C-terminal domain is present in the poles of mitotic spindles (not shown).

Depletion of GMAP210 induces fragmentation of the Golgi ribbon in isolated stacks that lost their pericentrosomal localisation but maintain normal secretion activity. This phenotype is reminiscent of that produced by MT depolymerisation and argues against a critical role of GMAP210 in protein transport. Instead, it provides support to our previous proposal that GMAP210 participates in Golgi ribbon formation around the centrosome. Depolymerisation of MTs is sufficient to generate ministacks indicating that Golgi ribbon structure normally undergoes dynamic lateral fusion processes depending on intact MTs. Data presented here support a model in which GMAP210 would participate in homotypic fusion of cis-cisternae by anchoring the surface of cisternae via its C-terminus and projecting its distal N-terminus to bind the rims or to stabilise tubular structures connecting neighbouring cis-cisternae. When fully extended, the long coiled-coil central domain of GMAP210 may achieve a length of approximately 200 nm, which might favour the tethering between cisternae. As a matter of fact, very short treatments with nocodazole (NZ), which was not yet producing Golgi scattering, induced a specific destabilisation of GMAP210 containing membranes that could correspond to MT-dependent intersaccular connections [10]. This model also agrees, to a large extent, with the molecular model proposed from *in vitro *studies [15], although the ALPS motif *in vivo *binds curved intra-Golgi membranes rather than vesicles.

The ability of GMAP210 to favour homotypic connection between stacks, coupled to its capacity to bind MT-minus ends, could provide a rationale to the recently reported capacity of GMAP210 to dock IFT20 on the GA [26]. During the transition from the centriole-based centrosome of cycling cells to the basal body-based primary cilium of post-mitotic cells, GMAP210 could re-orient part of the tethering and fusion processes from the cis face of the GA toward the basal body, mediating the coupling between axoneme assembly and ciliary membrane fusion and growth necessary to set the primary cilium as a new cell compartment.

## Conclusion

The Golgi localisation of GMAP210 is the result of the combined action of two distinct cis-membrane binding motifs that recognise different domains of the cis-GA. The N-terminal Golgi binding signal is localised at the first 38 residues of the protein corresponding to the ALPS motif, a lipid-binding module that folds into an α-helix once bound to curved membranes. It specifically accumulates at the periphery of cis-Golgi cisternae and in areas connecting adjacent stacks. The C-terminal domain, which includes the GRAB domain, also associates with the cis-cisternae but its distribution is continuous along the ribbon. Our data support a model in which GMAP210 would participate in homotypic fusion of cis-cisternae by anchoring the surface of cisternae via its C-terminus and projecting its distal N-terminus to bind the rims or to stabilise tubular structures connecting neighbouring cis-cisternae. In agreement with this model, depletion of GMAP210 induces fragmentation of the Golgi ribbon in isolated stacks that have lost their pericentrosomal localisation [9,13].

## Methods

### Antibodies

The autoimmune sera against GMAP210 and Golgin-245 (unpublished data) have been previously characterised. Rabbit anti-GMAP210 (RM130), rabbit anti-ninein polyclonal and mouse CTR433 monoclonal antibody have been produced and purified in our laboratories. Monoclonal antibody against giantin was kindly provided by HP Hauri (University of Basel/Switzerland). Monoclonal anti-GM130 and anti-Sec31 antibodies were from BD Biosciences (Erembodegen, Belgium) and polyclonal anti-giantin from Abcam (Cambrige, UK). Nocodazole (10 μM) and Brefeldin A (5 μg/ml) were purchased from Sigma (Madrid, Spain). Oligofectamine reagent and Lipofectamine 2000 reagent were from Invitrogen (Barcelona, Spain).

### Cell culture, cell extracts, electrophoresis and immunoblotting

These studies primarily used diploid, telomerase-immortalised human retinal pigment epithelial hTERT-RPE1 cells (Clontech, Mountain View, CA, USA), grown in DMEM-F12 medium. HeLa cells were grown under standard conditions. To generate a hTERT-RPE1 cell line with a fluorescent labelled GA, we used the pAcGFP1-Golgi vector, which encodes a fusion protein consisting of the N-terminal 81 amino acids of the human beta 1,4-galactosyltransferase, a membrane-anchoring signal peptide that targets the protein to the transmedial region of the GA, directly upstream of the green fluorescent protein from *Aequorea coerulescens *(GT_TM_-GFP). To improve the efficiency of selection, this sequence was subcloned into the bi-cistronic pIRESneo vector. The new construct was transfected in hTERT-RPE1 cells by using Lipofectamine and then selected with G418 for 4 to 6 weeks. Finally, cell population was sorted in a BD FACSaria cell-sorting system and only cells expressing high levels of the protein were maintained. Cell extract preparation, SDS-PAGE and immunoblotting procedures have been described previously [12].

### Molecular biology, transfection and siRNA

Truncated mutants were constructed in pGFP-C, pGFP-N or pDsRed-Express-N vector series by subcloning using internal sites or by PCR. All constructions were verified by sequencing. To rule out that the effect of small construct involving the amphipathic helix was due to adjacent sequences, different linkers were used. Sequences of GFP (in bold)-fusion proteins involving the amphipathic helix (italics) were as follows:

ALPS-GFPa: *MSSWLGGLGSGLGQSLGQVGGSLASLTGQISNFTKDML*STVPRARDPPVAT

### MVS

ALPS-GFPb: *MSSWLGGLGSGLGQSLGQVGGSLASLTGQISNFTKDML*DPPVAT**MVS**...;

ALPS2-GFP:*MSSWLGGLGSGLGQSLGQVGGSLASLTGQISNFTKDML*STVP*MSSWLGGLG*

*SGLGQSLGQVGG SLASLTGQISNFTKDML*PPVAT**MVS**...;

ALPS-CC(10pt)-GFP:*MSSWLGGLGSGLGQSLGQVGGSLASLTGQISNFTKDML*STLQQALS

DAENEIMRLSSLNQDNSLAEDNLKLKMRIEVLEKEKSLLSQEKEELQMSLLKLNNEYEVIK STATRDISLDSELHDLRLNLEKEQELNQSISEKETLIAEIEELDRQNQEATKHMILIKDQL SKQQNEGDSIISKLKQDLNDEKKRVHQLEDDKMDITKELDVQKEKLIQSEVALNDLHLTKQ KLEDKVENLVDQLNKSQESNVSIQKENLELKEHIRQNEEELSRIRNELMQSLKDPPVAT**MVS**...

RPE1 cells were transfected with Lipofectamine 2000 reagent and processed for IF 6 to 20 h after transfection. Cells were also transfected with small interfering RNA (siRNA) duplexes (Sigma, Madrid, Spain) and Oligofectamine according to the manufacturer's instructions. The sequence of the siRNA specific for the C-terminus of GMAP210 was 5'-UCAGCGUCAUGAAGUGUUA-3'. The sequence of the siRNA specific for the N-terminus of GMAP210 was 5'-GAAUGUCUUGCUGGUAACA-3'. After 48 or 72 h, cells were transfected with the Nter-GFP or the GFP-Cter constructs, incubated for an additional 16 h and then processed by IF.

### Immunofluorescence and live cell imaging

For immunofluorescence experiments, cells were grown on 12 to 15 mm round coverslips. Cells were fixed in 4% PFA during 10 min in PBS pH 7.4, permeabilised in PBS and 0.5% Triton and then labelled with appropriate antibodies. Cells were examined under a motorised upright wide-field microscope (Leica DM6000B) equipped for image deconvolution. Acquisition was performed at room temperature using an oil immersion objective (63× APO PL HCX 1.4 NA) and a cooled CCD camera (Leica DFC35FX). For confocal imaging, the samples were examined under a Leica SP5 confocal microscope. Optical sections were obtained with a 63× oil immersion objective (1.4 NA) at a definition of 1024 × 1024 pixels. For imaging of the fluorescent proteins in living cells, the transfected cells grown on coverslips were placed in a 37°C incubation chamber attached to a Leica DM6000B motorised microscope and filled with OptiMEM medium. Acquisition was performed using an oil immersion objective (63× APO PL HCX, 1.4 NA) and a cooled Hammamatsu camera. Z-positioning was accomplished by a piezo-electric motor. All images correspond to an optical section unless otherwise stated. Image analysis and 3D blind deconvolution was performed using the Leica and Adobe Photoshop and ImageJ 1.42j software. To obtain the intensity values along a given line, images were processed with Metamorph Offline 7.1.7.0 using the command linescan. To obtain a numerical measure of Golgi binding affinities of ALPS-CC-GFP or 1–76CC-GFP mutants, we determined the relative fluorescent intensities over the Golgi and the cytoplasm using the linescan function of the Metamorph software. Ten lines, containing 200 pixels each, were drawn at the Golgi or cytoplasm areas, respectively. Ten cells of each mutant were analysed.

## Abbreviations

ALPS: amphipathic lipid-packing sensor; GA: Golgi apparatus; GRAB domain: Grip-related Arf-binding domain; MT: microtubule.

## Authors' contributions

JC prepared constructions and executed most of the experiments. SR participated in some experiments. MB, BG and RMR conceived the study. RMR provided advice and overall direction, supervised the execution of the project and wrote the manuscript.

## Supplementary Material

Additional file 1 **Movie 1**. Dynamics of GMAP210 N-terminal domain in living cells. A RPE1 cell line stably expressing the transmembrane domain of galactosyltransferase fused to GFP (GT-GFP) was generated and transfected with the Nterminal domain of GMAP210 expressed in fusion with the DsRed protein (Nter-DsRed). Images were taken with 20-seconds intervals. Click here for file

Additional file 2 **Movie 2**. Live imaging of a GFP-Cter transfected cell during treatment with BFA. BFA was added to a final concentration of 2.5 μg/ml at time 5.0 seconds and images were taken with 0.2-second intervals. Click here for file

Additional file 3 **Movie 3**. Dynamics of a RPE1 cell co-transfected with both Nter-DsRed and GFP-Cter fusion proteins. Images were acquired every 5 minutes. The duration of the movie is around two hours. Click here for file

Additional file 4 **Movie 4**. Live imaging of a RPE1 cell co-transfected with both Nter-DsRed and GFP-Cter fusion proteins during nocodazole treatment. Images were acquired every 5 minutes. The duration of the movie is around two hours. Click here for file
